# Obituary – Luiz Carlos Severo (1948-2022)

**DOI:** 10.1016/j.bjid.2022.102382

**Published:** 2022-07-02

**Authors:** Flavio de Mattos Oliveira, Alessandro C. Pasqualotto

**Affiliations:** a Santa Casa de Misericórdia de Porto Alegre, Porto Alegre, RS, Brazil; b Universidade Federal de Ciências da Saúde de Porto Alegre, Porto Alegre, RS, Brazil

Dear Editor,

**Figure:**
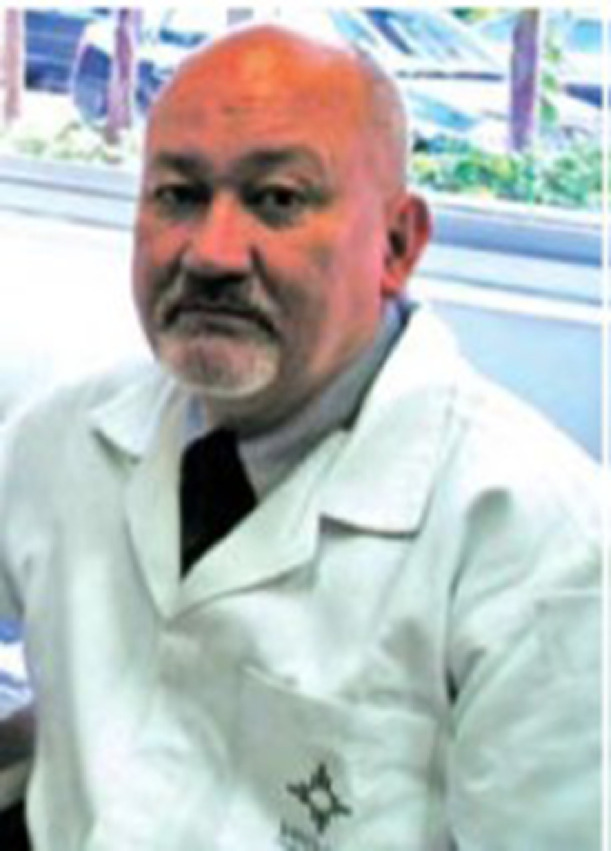
Dr Severo at Santa Casa de Misericórdia de Porto Alegre

Severo was born into a simple family in countryside (Cachoeira do Sul, RS). Initially, he was interested in the agrarian area, as he loved to plant. However, it was his passion for books that led him to study Medicine at the Federal University of Santa Maria (1976), home of renowned professor Alberto T. Londero. Still in the 2^nd^ year of medical school, Severo failed Londero's course of Parasitology, which made him study really hard, and to develop a growing admiration for his “master” – a word that Severo used quite frequently and with affection when referring to his professor. Londero and Severo had a relationship that lasted for many years, always fruitful and sometimes interrupted by periods of disagreements. Londero was also of great importance by presenting Severo to Nelson Porto, an important radiologist at the Santa Casa de Porto Alegre, a hospital at which Severo worked until his late days. Severo took his master by example, and became a very methodical man. He was always telling jokes – a scientist with an acid humour, sometimes difficult to get along with, but always followed by faithful friends.

He started his professional career as a lung physician, working at the Partenon Sanatorium Hospital. There, he noticed that several cases of mycoses were erroneously diagnosed as tuberculosis. In order to better study such cases, Severo quite often took the night train to Santa Maria to discuss cases and confirm diagnoses with his master. With the support of the health state secretary Germano Bonow, Severo created the city's first Mycology Laboratory in Porto Alegre, at the former Biological Research Institute. The Santa Casa Mycology Laboratory became known for diagnosing difficult acses, for the organization of its cases, and for the exuberant scientific production of the group. Throughout his career, Severo trained many people in mycology, including 40 masters and doctors. He has authored 80 book chapters and presented around 300 abstracts in scientific conferences. He has published over 250 scientific articles, most of them written on a bar table. He definitely enjoyed his life.

Severo studied pretty hard throughout his life. He obtained a Master's degree in Pulmonology from the Federal University of Rio Grande do Sul (UFRGS, 1979). Subsequently, a PhD in Medicine at UFRGS (1987) and post-doctorate in immunochemistry of aspergillosis by the Public Health Laboratory Service, UK (1989). He became a full professor in Infectious and Parasitic Diseases at the FFFCMPA (1993; today UFCSPA). He was, for many years, a level 1 researcher at the National Council for Scientific and Technological Development, and a professor at the UFRGS. More recently, he dedicated himself to the study of philosophy, having obtained a Masters in Philosophy from the Pontifical Catholic University of RS (2003). He had Nietzsche as his greatest philosopher, and he did not tolerate theological thoughts.

Severo was a very complete mycologist, one of the few in the field. He knew pretty much about the clinical aspects of mycoses, as he had a great understanding of pulmonology, radiology, and laboratory practices. He has done research in many areas of mycology, including endemic mycoses, opportunistic fungal infections, actinomycosis and subcutaneous mycoses.

With his departure, the world not only loses a great scientist, a man of free thinking who was not afraid to expose his contradictions to society. It also loses a great educator, a philosopher and a lover of mycology. Severo will be missed by family, friends, and students, and we hope that his followers can expand the beauty of his work.

## Conflicts of interest

The authors declare no conflicts of interest.

